# A Novel Computational Method to Reduce Leaky Reaction in DNA Strand Displacement

**DOI:** 10.1155/2015/675827

**Published:** 2015-09-30

**Authors:** Xin Li, Xun Wang, Tao Song, Wei Lu, Zhihua Chen, Xiaolong Shi

**Affiliations:** ^1^Department of Obstetrics and Gynecology, Renmin Hospital of Wuhan University, Wuhan, Hubei 430060, China; ^2^College of Mathematics and System Science, Shandong University of Science and Technology, Qingdao, Shandong 266590, China; ^3^College of Computer and Communication Engineering, China University of Petroleum, Qingdao, Shandong 266580, China; ^4^Faculty of Engineering, Computing and Science, Swinburne University of Technology Sarawak Campus, 93350 Kuching, Sarawak, Malaysia; ^5^Key Laboratory of Image Information Processing and Intelligent Control, School of Automation, Huazhong University of Science and Technology, Wuhan, Hubei 430074, China

## Abstract

DNA strand displacement technique is widely used in DNA programming, DNA biosensors, and gene analysis. In DNA strand displacement, leaky reactions can cause DNA signals decay and detecting DNA signals fails. The mostly used method to avoid leakage is cleaning up after upstream leaky reactions, and it remains a challenge to develop reliable DNA strand displacement technique with low leakage. In this work, we address the challenge by experimentally evaluating the basic factors, including reaction time, ratio of reactants, and ion concentration to the leakage in DNA strand displacement. Specifically, fluorescent probes and a hairpin structure reporting DNA strand are designed to detect the output of DNA strand displacement, and thus can evaluate the leakage of DNA strand displacement reactions with different reaction time, ratios of reactants, and ion concentrations. From the obtained data, mathematical models for evaluating leakage are achieved by curve derivation. As a result, it is obtained that long time incubation, high concentration of fuel strand, and inappropriate amount of ion concentration can weaken leaky reactions. This contributes to a method to set proper reaction conditions to reduce leakage in DNA strand displacement.

## 1. Introduction

DNA is increasingly being regarded as an excellent nanoscale engineering material due to its functionality and programmability. DNA nanotechnologies have been developed to create complex synthetic biomolecular circuits [1, 2], DNA-based nanodevices [3], machines [4], and biosensors [5]. Of particularly widely used techniques in the DNA programming, DNA biosensors and gene analysis are the DNA strand displacement technique [6–10] which is both cell-free and enzyme-free and allows DNA-based signal processing and amplification merely according to specific nucleic acid sequences [7, 11, 12]. DNA strand displacement with Fuel strands and toehold exchange process can embody digital logical gates and can be integrated into programmable logical computational circuits [13–17]. Such technique has also been integrated with DNA tile based self-assembly to adjust their internal changes and to “smartly” interact with their surroundings [18, 19]. Although DNA strand displacement performs significantly in many fields, there are still some challenging problems. One of the most bothersome defects is the leaky reaction.

**Figure 1:**
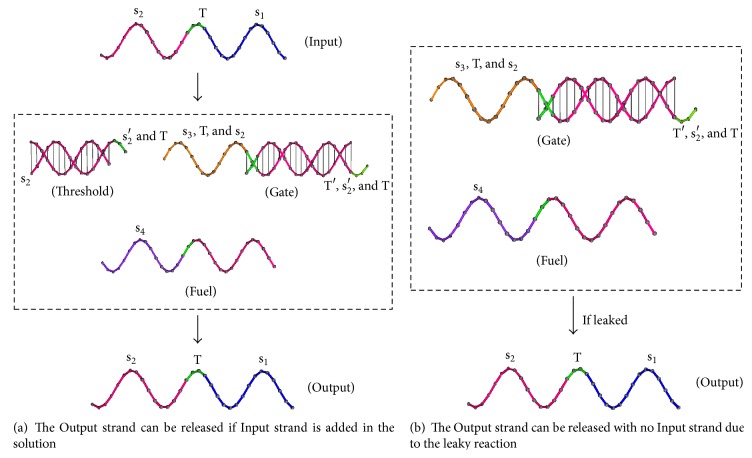
An example of leaky reaction.

In DNA strand displacement, the toehold exchange process is a fast and reversible strand displacement reaction. There is a unique exposed region in a partially double-stranded DNA molecule, and only the DNA signal strand with a region complementary to the region can initiate strand displacement and release another DNA signal strand. But practically, the target outputting strands could be issued without any input DNA signal strand, which is called leaky reaction. An example of leaky reaction is shown in Figure 1. Leaky reaction can cause DNA signals decay and detecting target DNA signals fails, thus resulting in logic errors in DNA strand displacement chain reactions.

In previous works, the mainly used method to avoid leakage is cleaning up after upstream leaky reactions [7, 8, 13, 14], and in recent years some other methods have been developed to avoid the leakage or to reduce the influences it brings. In [20], Soloveichik et al. proposed a method to ameliorated the leakage by providing auxiliary complexes at low concentrations in a continuous-flow stirred-tank reactor. For particular applications, Fontana provided a toolkit of DNA-based reactions for digital signal processing, which includes thresholds to remove leaks and amplifiers to restore signal strength [21]; Qian et al. employ Threshold complex and add an extra signal restoration step consisting of an integrating gate and an amplifying gate before reporting processes to suppress leakage [15].

The main idea of the above methods is using extra reactions to remove leakage (products of leaky reactions). It remains a challenge to develop reliable DNA strand displacement by reducing the leakage with setting proper reaction circumstances. In [22] the effect of the variation of reaction temperature on leaky reaction was studied, but there are several other elemental factors that can cause leaky reactions. In DNA strand displacement shown in Figure 1(a), the two DNA strands forming the Gate complex may not bind together completely after annealing and the double-stranded region of DNA may fray slightly at one end forming a short exposure of one strand that can function as a toehold. With such exposed region, Fuel strands may bind upon Gate strand, thus initiating a strand displacement and producing the Output strand with no Input strands present. Gate complex with unexpected exposed regions can be with a very small proportion of all reactants. The rate of leakage reaction constants up to a much slower ration is compared to the ordinary strand displacement reaction with certain reaction time. Hence, it gives some hints to support that reaction time and the concentration of the Fuel strands are two crucial factors for leaky reaction.

In this work, we experimentally evaluate the influences of reaction time, ratio of reactants, and ion concentration to the leakage in DNA strand displacement, and then mathematical models for calculating the leakage in DNA strand displacement are achieved by experimental curve derivation. In the experiments, fluorescent probes and a hairpin structure reporting DNA strand are designed to detect the output of DNA strand displacement and thus can be used to evaluate the leakage of DNA strand displacement in different reaction time, ratios of reactants, and ion concentrations. Experimental results are analyzed by PAGE gel electrophoresis for qualitative analysis and qRT-PCR for quantitative analysis. It is obtained that long time incubation, high concentration of Fuel strand, and inappropriate amount of ion concentration can weaken leaky reaction. The mathematical model of leakage in DNA strand displacement reactions is highly congruent with the experimental observations. This contributes to a method to reduce leakage in DNA strand displacement by setting proper reaction conditions.

## 2. Materials and Methods

### 2.1. Preparation of the Experiments

#### 2.1.1. The Logic System and Oligonucleotide Sequences

Nine different DNA strands with reusable domains were designed for the experiments. Sequences of the strands form P1 to P9 and reusable domains are listed in Tables 1 and 2. Component samples, which are constructed by Strands T1, T2, T3, T4, T5, and T6, and the function of each sample are depicted in Table 3.

We set up two groups of comparing experiments, namely, Group A and Group B. Each experiment in Group A includes Input, Threshold, Gate, and Fuel, of which the output can be produced according to components circumstances, while the experiments in Group B (without Input) consist of Gate and Fuel, and the output can be produced in case leaky reactions happened. The two groups of experiments have their own Reporter strands to detect the output in DNA strand displacement.

**Figure 2:**
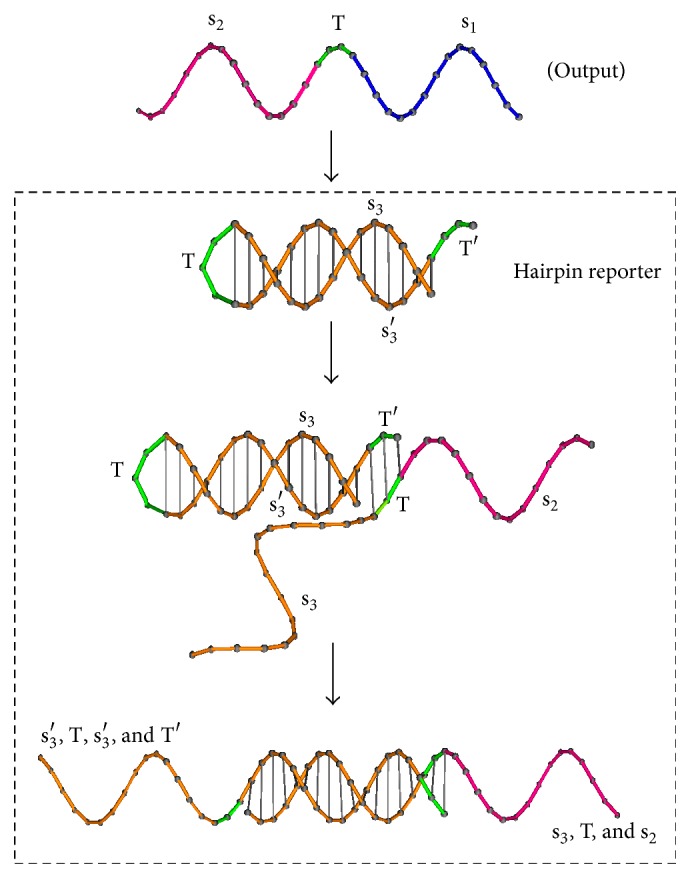
Reporter strand with “hairpin” to detect whether Output strands are being released.

In experiments in Group A, a hairpin structure is designed to be detected as the variety of its length by PAGE gel electrophoresis. Adhering the uncovered toehold “T” of the hairpin structure, the output will compete with the stem structure, stretching the hairpin structure as longer strands; see Figure 2. Output strands will adhere to Reporter strand and unfold its hairpin structure, which is called reporting reaction in the PAGE analysis.

**Figure 3:**
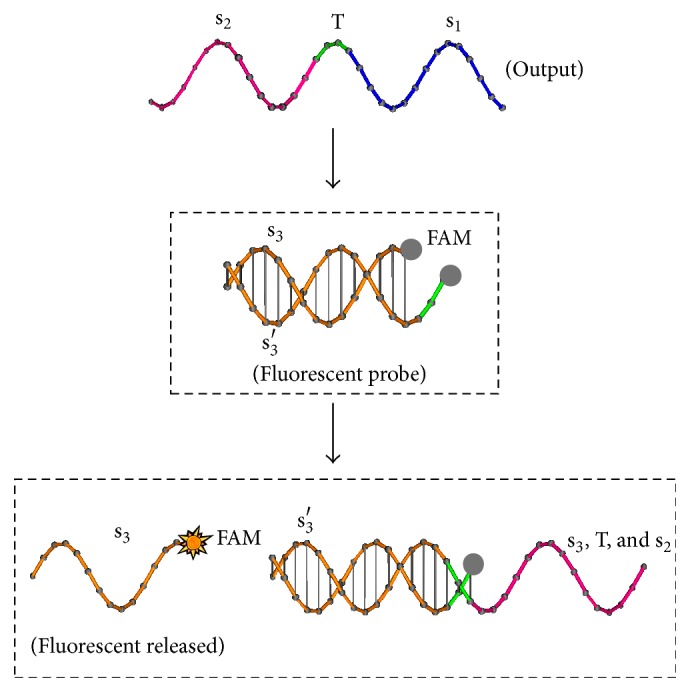
Fluorescent probe is set for reporting the output in qRT-PCR.

In experiments in Group B, a fluorescent probe will be used to detect the amount of outputs according to the fluorescence intensity. When the Output strand is released, the probe strands will be displaced and the fluorescent strand will be revealed and detected; see Figure 3. The qRT-PCR will be used to detect the fluorescence intensity cycle by cycle.

#### 2.1.2. DNA Oligodeoxynucleotide Strands

The DNA strands are ordered from Sangon Biotech (Shanghai, China) Co., Ltd. The DNA strands, 3′-labeled with fluorophore 6-FAM (FITC) and 5′-labeled with corresponding quenchers, are purified by HPLC, while all the others are with PAGE purification.

#### 2.1.3. Reagents and Equipment

Ultrapure water is used for every mixture. 0.5x TAE buffer consists of 20 mM Tris (pH 7.6), 1 mM EDTA, and 10 mM acetic acid. The standard concentration of Mg^2+^ solution is 25 mM magnesium acetate. The RT PCR proceeding the experiments of Group B is bought from Xi'an TianLong Science and Technology Co., Ltd. The equipment of gel electrophoresis is made by Beijing Liuyi Instrument Factory.

### 2.2. Experimental Process

*The Experiments in Group A*

(A1) Set up the double strands. We design three kinds of double strands in the experiments: T2 formed by P2 and P3; T3 formed by P4 and P5; and T5 formed by P7 and P8. Each of the solutions contains 4 *μ*M strand Pa and 4 *μ*M strand Pb with 0.5x TAE/Mg^2+^ buffer. All the solutions are incubated at 95 for 3 minutes and then cooled down to 4°C over 16 hours.
(A2) The concentrations of sample T1 in three of four different mixtures are 20 nM, 40 nM, and 80 nM, respectively, and the other one is blank. All the mixtures are including 40 nM T2, 40 nM T3, 80 nM T4, 80 nM T5, and 25 *μ*M Mg^2+^ with 0.5x TAE, incubated at 25°C for 16 hours.
(A3) Each of the four mixtures contains sample T4 with 80 nM, 160 nM, 80 nM, and 1.28 *μ*M, respectively; with 40 nM T3, 80 nM T5, 25 *μ*M Mg^2+^, and 0.5x TAE. The samples are kept in the qRT-PCR at 25°C for 16 hours.
(A4) There are four mixtures consisting of 40 nM T3, 80 nM T4, and 80 nM T5 in 0.5x TAE. Each sample has different concentration of Mg^2+^, respectively, zero, 25 *μ*M, and 100 *μ*M, and incubates at 25°C for 16 hours.

*Experiments in Group B*

(B1) All primers labeled from P1 to P7 and P9 are dissolved with 1x TAE buffer and set the concentration of every single-strand DNA to 100 *μ*M. The concentrations of Input, Gate, Threshold, and Reporter strands are set to 5 *μ*M, while the concentration of Fuel strand is set to 10 *μ*M.
(B2) All the experiments in this group are incubated at 20°C. The Reporter strand is added and incubated for another 0.5 hours.
(B3) The mixture of the Output and Reporter strands and merely the Reporter strands are incubated with other samples in the same conditions.
(B4) After the same reporting process, the results of all experiments are analyzed by nondenaturing polyacrylamide gel electrophoresis (PAGE) at room temperature. The concentration of relatum is about 20% and the voltage of electrophoresis is 80 V.

**Figure 4:**
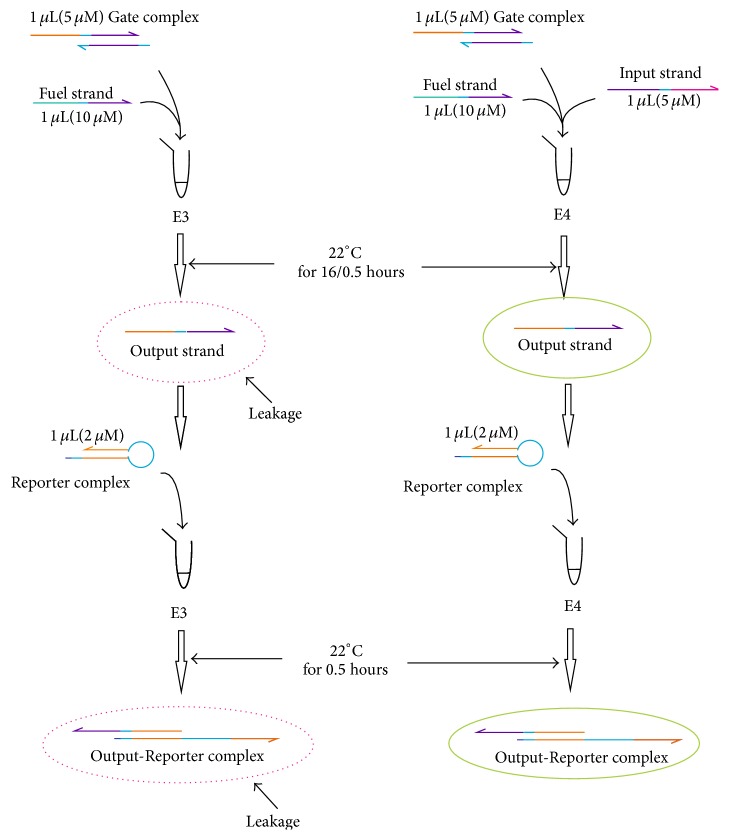
The procedures of experiments to analyze the influence of reaction time (16/0.5 hours) to leaky reaction of DNA strand displacement.

The procedures of experiments to analyze the influence of reaction time (16/0.5 hours) to leaky reaction of DNA strand displacement are shown in Figure 4, which corresponds to experiment (B2) in Group B.

**Figure 5:**
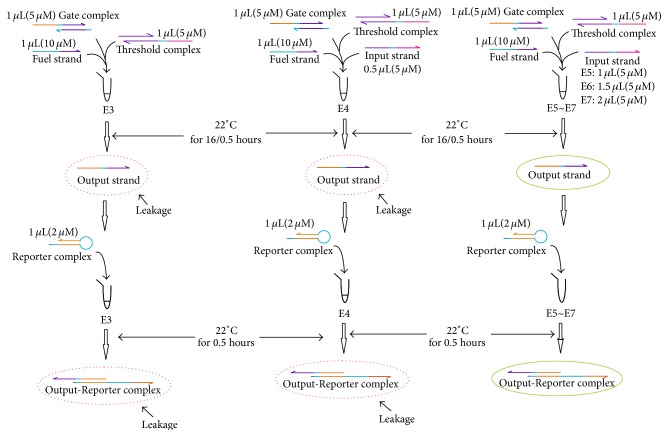
The procedures of experiments to analyze the influence of reaction time (16/0.5 hours) to leaky reaction of DNA strand displacement with Threshold strands.

**Figure 6:**
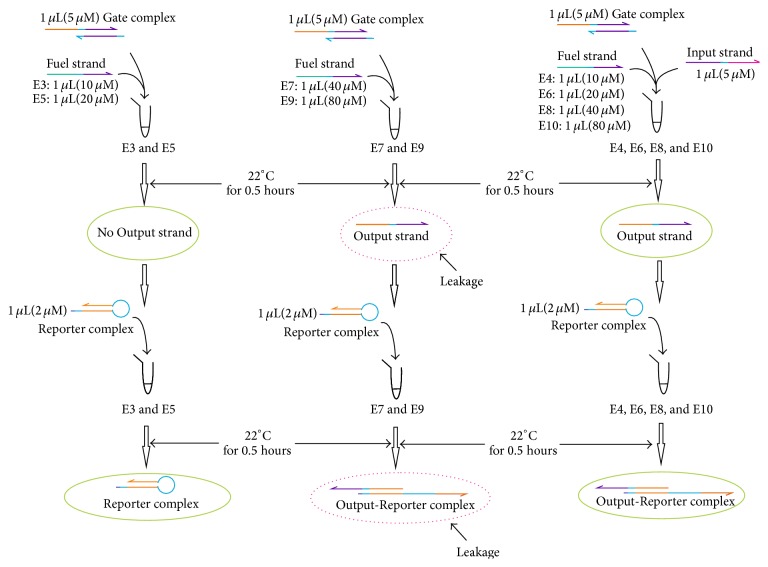
The procedures of experiments to analyze the influence of reaction time (16/0.5 hours) to leaky reaction of DNA strand displacement with different concentration of Fuel strands.

The procedures of experiments to analyze the influence of reaction time (16/0.5 hours) to leaky reaction of DNA strand displacement with Threshold strands and different concentration of Fuel strands, corresponding to experiments (B2) and (B3) in Group B, are given in Figures 5 and 6, where the components mixed with specific concentrations and their incubated times are specified.

## 3. Results

### 3.1. Experimental Results

**Figure 7:**
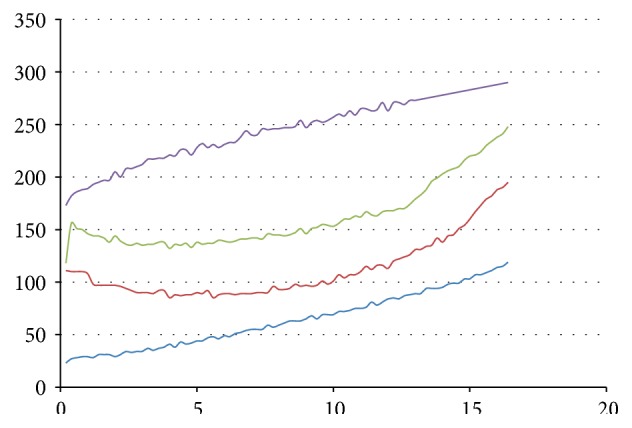
The fluorescent intensity of Output strands from experiment (A1). *x*-axis is reaction time and *y*-axis is the fluorescent intensity of 6-FAM. From bottom up, the curves correspond to cases of concentrations of Input strand, being 0 nM (blue), 20 nM (red), 40 nM (green), and 80 nM (purple), respectively.

**Figure 8:**
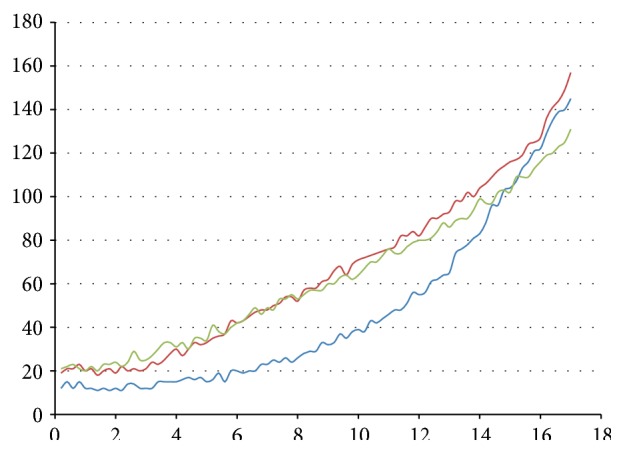
The fluorescent intensity of Output strands from experiment (A2). *x*-axis is reaction time and *y*-axis is the fluorescent intensity of 6-FAM. From bottom up, the curves correspond to cases of concentrations of Fuel strands being 80 nM (blue), 160 nM (green), and 1.28 *μ*M (red), respectively.

**Figure 9:**
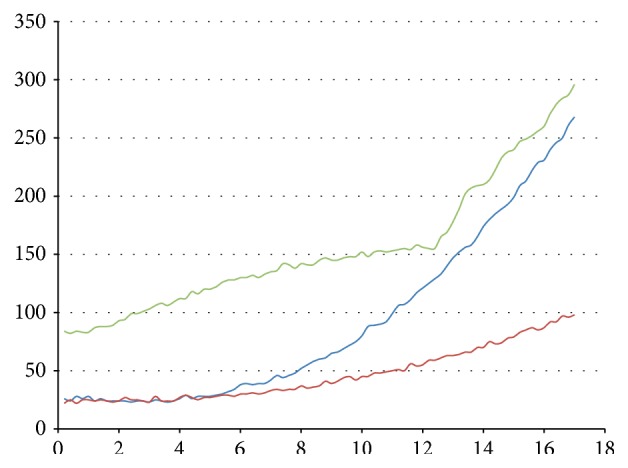
The fluorescent intensity of Output strands from experiment (A3). *x*-axis is reaction time and *y*-axis is the fluorescent intensity of 6-FAM. From bottom up, the curves correspond to cases of concentrations of magnesium ion, being 0 (blue), 25 *μ*M (red), and 100 *μ*M (green), respectively.

**Figure 10:**
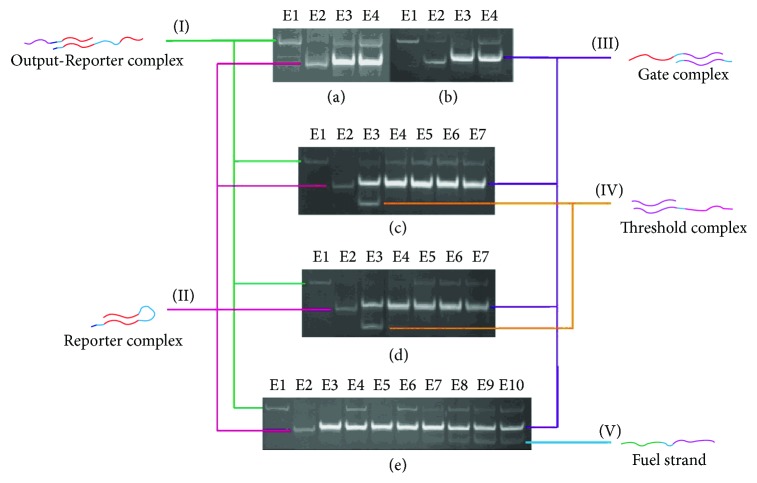
PAGE results of all experiments; identities of all samples are on the top of the gel.

(i) In experiments in Group A, Reporter strand T5 with a fluorescent probe (see Figure 3) is used to detect whether Output strand is released. The higher the concentrations of Output strands are, the greater the fluorescent intensity will be. In experiment (A1), we set strand P1 as Input, strand T2 as Threshold, strand T3 as Gate, and strand P6 as Fuel. The fluorescent intensity of Output strands from experiments (A1) is shown in Figure 7, where four cases with different concentrations of the Input strands, 0, 20 nM, 40 nM, and 80 nM, are tested. It is obtained that, with the concentration of the Input strands becoming large, the fluorescent intensity of Output strands becomes large.
 In experiments (A2) and (A3), we set strand T3 as Gate and strand P6 as Fuel. In the experiments, the leakages in DNA strand displacement with different concentrations of Fuel strand and ion are compared, respectively; see Figures 8 and 9. In Figure 8, fluorescent intensities of Output strands in DNA strand displacement with different concentration of Fuel strands, 80 nM, 160 nM, and 1.28 *μ*M, are shown, while, in Figure 9, fluorescent intensities of Output strands with concentrations of magnesium ion being 0, 25 *μ*M and 100 *μ*M, respectively, are given.
(ii) In experiments in Group B, hairpin structure T6 is set to be the reporter of Output strands (see Figure 2). Input strands are set as a contrast for showing the result in the PAGE analysis. In Figure 10 the PAGE analysis of experiment results and their identities of all the bands of all the samples are shown. Each sample E1 and E2 in Figure 10 is the mixture of the Output&Reporter strands and merely the Reporter strands, respectively. In experiment (B2), leakage in DNA strand displacement with different reaction times (see Figures 10(a) and 10(b)) is compared with the leakage with different concentrations of Input strands; see Figures 10(c) and 10(d). In experiment (B3), leakage in DNA strand displacement is compared with different concentrations of Fuel strands (Figure 10(e)).

In Figure 10, (a) is experimental results of the influence of reaction time to leaky reactions with seesawing reaction taking 16 hours and the reporting reaction taking 0.5 hours; (b) is the cases of DNA strand displacement reaction taking 0.5 hours and the reporting reaction taking 0.5 hours; (c) is experimental results of the influence of reaction time to leaky reactions with Threshold strands with DNA strand displacement reaction taking 16 hours and the reporting reaction taking 0.5 hours; (d) is the case of seesawing reaction with threshold taking 0.5 hours and the reporting reaction taking 0.5 hours; and (e) is experimental results of the influence to leaky reactions with different concentrations of Fuel strands, where seesawing reaction takes 0.5 hours and reporting reaction takes 0.5 hours. Samples E3 and E4, E5 and E6, E7 and E8, and E9 and E10 are four small control groups with different concentrations of Fuel strand. Samples E4, E6, E8, and E10 are incubated with Input strands, while samples E3, E5, E7, and E9 are not.

### 3.2. Derived Mathematical Model

#### 3.2.1. Mathematical Model of Leakage and Reaction Time

For experiments in Group A, we use MATLAB to analyze the mathematical model of leaky reactions in the DNA strands displacement process. Model-fitting analysis of time series of the fluorescent intensity data is conducted, and the models of leakage based on reaction conditions, including reaction time, ratio of reactants, and ion concentration, are set up. Generally, in the following models, parameter *x* represents reaction time with unit hour and *f*(*x*) denotes the fluorescent intensity in proportion in the produce of output in DNA strands displacement.

From experiment (A1) in Group A, the leaking equation with different concentrations of Input strands can be derived by data fitting analysis, which is of the form

(1) $$\begin{array}{c} f\left(\;x\;\right)=\left\{\begin{array}{cc} 100n\times e^{x\cdot \ln x/100n\left|\ln x\right|}+30e^{0.1x}, & n>0;\\ 30e^{0.1x}, & n=0, \end{array}\right. \end{array}$$

where *n* is the amount of multiples of Input strands, with one multiple being 40 nM. The fluorescent intensity is on the rise with growth of the multiples of Input strands, as well as the trends of the fluorescent intensity in the four solutions increased over the time. With this fact, we have

(2) $$\begin{array}{c} f^{\prime }\left(\;x\;\right)=\frac{df\left(x\right)}{dx}=\left\{\begin{array}{cc} 3e^{0.1x}-e^{-x/100i}, & 0\leq i<1;\\ 3e^{0.1x}+e^{x/100i}, & i\geq 1. \end{array}\right. \end{array}$$

Since 3*e* ^0.1*x*^ ≫ *e* ^−*x*/100*i*^ with 0 ≤ *i* < 1 and 3*e* ^0.1*x*^ ≫ *e* ^*x*/100*i*^ with *i* ≥ 1, the model can be simplified as

(3) $$\begin{array}{c} f^{\prime }\left(\;x\;\right)=3e^{0.1x},\;x\geq 0. \end{array}$$

Also, it can be further derived as

(4) $$\begin{array}{c} f^{\prime \prime }\left(\;x\;\right)=\frac{df^{\prime }\left(x\right)}{dx}=0.3e^{0.1x},\;x\geq 0. \end{array}$$

From the obtained model, it is found that the variance and the ratio of Output strands are dependent with parameter *i*, which means that different concentrations of Input strands have the same tendency of Output strands and the reaction time is exponential with respect to the encasement of multiples of Input strands. Due to the fact that fluorescent intensity is proportional with respect to Output strands, it is obtained that the appropriate reaction time is 2.5 hours, which can control the increment of leakage under 1%.

#### 3.2.2. Mathematical Model of Leakage and Concentration of Fuel Strands

The mathematical model of leakage and concentration of Fuel strands can be obtained by data fitting analysis from experiments (A2). The obtained model is as follows:

(5) $$\begin{array}{c} f\left(\;x\;\right)=\frac{24i-22}{i-0.7}\times e^{\left(0.261-0.065i\right)x},\;i\geq 1. \end{array}$$

The unit concentration of Fuel strand is denoted by 2× being 80 nM, and by parameter *i* we denote the power of 2× (2^*i*^×). The curve of model is concave down. The leakage increases when *i* ≤ 4, and the rate of increase is up with the growth of Fuel strands concentration. When *i* > 4, the leakage presents the trend of decreasing. It shows that, in high concentration of Fuel strands, all DNA strands in the solution are quick balanced. High content of DNA strands makes the fluorescence quench each other, performing the downtrends and disrupting the rule of DNA strands displacement. This is highly congruent with the observations in experiment (A2).

#### 3.2.3. Mathematical Model of Leakage and Concentration of Ion Magnesium

The mathematical model of leakage and concentration of ion magnesium can be obtained by data fitting analysis from experiment (A3). It is obtained as

(6) $$\begin{array}{c} f\left(\;x\;\right)=12\times e^{\left(0.06c+3\right)/\left(c+16\right)+0.02c},\;c\geq 0, \end{array}$$

where *c* stands for the concentration of magnesium ion with unit *μ*M. From the equation, it is indicated that the leakages are enhancing with time. It can be calculated that when *c* ≈ 25, the fluorescent intensity is the weakest one at any point of time, which is the most suitable concentration of magnesium ion in DNA strands displacement. The mathematical result is highly congruent with the observations in experiment (A3).

### 3.3. Results by PAGE

In experiments in Group B, some qualitative results of PAGE are analysed below on the basis of Figure 10.

In Figures 10(a) and 10(b), the results of experiments without Threshold complex are shown. Patterns in lane 4 are almost the same in both experiments, but there is an appreciable difference in lane 3 between the two experiments. A luminous upper band in the third lane in Figure 10(a) indicates that Output strand is released with no input present; contrarily, there is almost no strand in the same position in Figure 10(b). This indicates that leaky reaction occurs easily in experiments with long time incubation, comparing with experiments with relatively short time incubation.

In Figures 10(c) and 10(d), the results of experiments with Threshold complex are shown. Patterns from lane 3 to lane 7 in Figure 10(c) are almost the same, indicating Output strands exiting in the samples. In Figure 10(d), patterns in lanes 3 and 4 are different from lane 5 to lane 7, which indicates that few Output strands are released in the case that the concentration of Input strands is significantly less than that of Threshold complex. It is indicated by lane 5 that when the volume of the Input strands equals that of Threshold complex, the Output strand will also be released. Therefore, it is concluded that when the DNA strand displacement reaction takes too long time, the leaky reaction accompanies it, but it can be avoided efficiently by reducing the reaction time.

In experiment (B3) (the procedure is shown in Figure 6), different concentrations of the Fuel strands are used. It is observed that patterns of lanes 1, 4, 6, 8, and 10 in Figure 10(e) are almost the same. Patterns of lanes 3, 5, 7, and 9 demonstrate the difference level of leakage. The upper bands become more and more luminous from lane 3 to lane 9, which indicates that the more the Fuel strands added, the more the Output strands released. We can conclude that Fuel strand promotes leaky reaction; that is, too many Fuel strands will lead to many leaks. An advisable concentration of Fuel strands is twice as much as that of the Output strands according to our experiments observations.

A notable fact obtained from PAGE analysis is that even when the reaction time is tailored and concentration of Fuel strands is reduced, the leaky reaction still exists and a small quantity of Output strands will be produced, particularly in Figures 10(b) and 10(d). It is possible that Input strand impinges Gate complex before it is absorbed by Threshold complex, and then seesawing reaction can be initiated to produce Output strand as a result of leak. But this kind of leaky reaction occurs with very low probability and can be negligible in most cases. It is noteworthy that the concentrations of components in our experiments are much higher than previous works, and the reaction time is much shorter than theirs. So we can conclude that the reaction time of DNA displacement is in certain functional relation with concentrations of reactants, and high concentrations lead to fast reactions and severe leakage.

## 4. Discussion

In this work, we have experimentally evaluated the influence on leaking in DNA strands displacement with different reaction conditions, including reaction time, the proportion of input with threshold, the proportion of fuel, and ion concentration. In the experiments, fluorescent probes and a hairpin structure reporting DNA strand are designed to detect the output of DNA strand displacement, that is, to evaluate the leakage. Experimental results are analyzed by PAGE gel electrophoresis (for qualitative analysis) and qRT-PCR (for quantitative analysis). As a result, it is concluded that long time incubation, high concentration of Fuel strand, and inappropriate amount of ion concentration can weaken leaky reaction. By data fitting analysis, we obtain mathematical models for calculating the leakage. The obtained results can contribute to a method to reduce leakage in DNA strand displacement, which is achieved by setting proper reaction conditions and using suitable amount of specific strands.

## Figures and Tables

**Table 1:** Sequences of the DNA strands.

Name	Domains	Sequence (5′-3′)
P1	s_2_ + T + s_1_	CACCACCAAACTTCATCTCACCCTAAAATCTCA
P2	s_2_	CACCACCAAACTTCA
P3	T′ + s_2_′	AGATGAAGTTTGGTGGTG
P4	s_3_ + T + s_2_	CACTAACATACAACATCTCACCACCAAACTTCA
P5	T′ + s_2_′ + T	AGATGAAGTTTGGTGGTGAGA
P6	s_4_ + T + s_2_	CAACATATCAATTCATCTCACCACCAAACTTCA
P7	T′ + s_3_′	AGATGTTGTATGTTAGTG
P8	s_3_	CACTAACATACAACA
P9	T′ + s_3_′ + 15T + s_3_	AGATGTTGTATGTTAGTG-TTTTTTTTTTTTTTT-CACTAACATACAACA

**Table 2:** Components samples.

Name	Sequence (5′-3′)	Length
T1	TCT	3
s_1_	CACCCTAAAATCTCA	15
s_2_	CACCACCAAACTTCA	15
s_3_	CACTAACATACAACA	15
s_4_	CAACATATCAATTCA	15

**Table 3:** Domains of the DNA strands.

Sample ID	Function	Formation
T1	Input	P1
T2	Threshold	P2, P3
T3	Gate	P4, P5
T4	Fuel	P6
T5	Reporter	P7, P8
T6	Reporter	P9
